# Enteric Glial Cells in Immunological Disorders of the Gut

**DOI:** 10.3389/fncel.2022.895871

**Published:** 2022-04-28

**Authors:** Chang Liu, Jing Yang

**Affiliations:** ^1^Center for Life Sciences, Peking University, Beijing, China; ^2^Academy for Advanced Interdisciplinary Studies, Peking University, Beijing, China; ^3^College of Life Sciences, Wuhan University, Wuhan, China; ^4^State Key Laboratory of Membrane Biology, School of Life Sciences, Peking University, Beijing, China; ^5^IDG/McGovern Institute for Brain Research, Peking University, Beijing, China; ^6^Chinese Institute for Brain Research, Beijing, China; ^7^Institute of Molecular Physiology, Shenzhen Bay Laboratory, Shenzhen, China

**Keywords:** enteric glial cells, enteric nervous system, inflammatory bowel disease, celiac disease, autoimmune enteropathy

## Abstract

Enteric glial cells (EGCs) are one of the major cell types of neural crest lineage distributed in the gastrointestinal tract. EGCs represent an integral part of the enteric nervous system (ENS) and significantly outnumber ENS neurons. Studies have suggested that EGCs would exert essential roles in supporting the survival and functions of the ENS neurons. Notably, recent evidence has begun to reveal that EGCs could possess multiple immune functions and thereby may participate in the immune homeostasis of the gut. In this review article, we will summarize the current evidence supporting the potential involvement of EGCs in several important immunological disorders, including inflammatory bowel disease, celiac disease, and autoimmune enteropathy. Further, we highlight critical questions on the immunological aspects of EGCs that warrant future research attention.

## Introduction

Enteric glial cells (EGCs) represent an indispensable component of the enteric nervous system (ENS; Gabella, 1972). EGCs and the ENS neurons are both derived from the neural crest during embryonic development (Nagy and Goldstein, 2017; Rao and Gershon, 2018). Notably, EGCs significantly outnumber the ENS neurons in adulthood, i.e., approximately 6:1 in the human myenteric plexus (Hoff et al., 2008). Similar to other glial cells in the central and peripheral nervous systems, e.g., astrocytes and Schwann cells, EGCs have essential roles in maintaining the survival and functions of surrounding neurons (Abdo et al., 2010). Of importance, recent studies have increasingly suggested that EGCs could also exert multiple immune functions and potentially modulate immunological disorders and other disease conditions of the gastrointestinal tract (Ibiza et al., 2016; Kermarrec et al., 2016; Chow et al., 2021; Progatzky et al., 2021). Here, we will first briefly overview the development and classification of EGCs and then focus on their emerging immune functions.

## Development and Classification of EGCs

**Figure 1:**
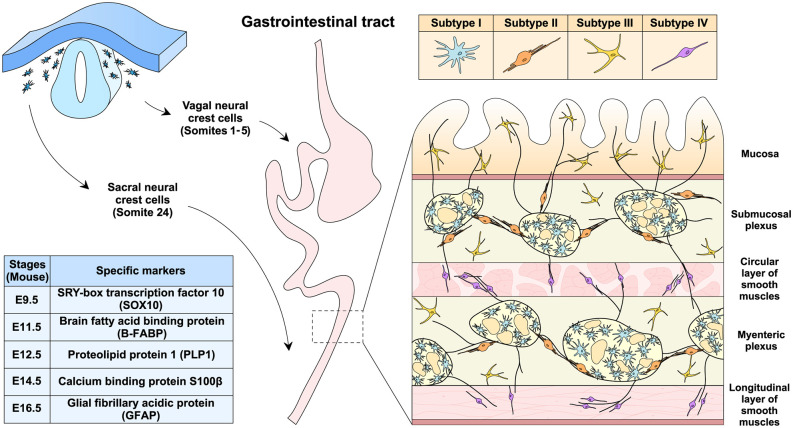
EGCs development, distribution, and classification. EGCs originate from the vagal (somites 1–5) and sacral (somite 24) neural crest cells during embryonic development in the mouse. Progenitor cells of EGCs sequentially express the specific markers, including SOX10 at E9.5, B-FABP at E11.5, PLP-1 at E12.5, S100β at E14.5, and GFAP at E16.5. In the gastrointestinal tract of adult mice, EGCs could be classified into subtypes I to IV based on cellular morphology and distribution within different anatomical layers. As an integral part of the enteric nervous system, EGCs are present both inside and outside the submucosal and myenteric plexus.

The primary origin of the ENS is vagal neural crest cells that migrate into the gastrointestinal tract from the rostral foregut to the caudal hindgut (Figure 1). In the mouse embryos, vagal neural crest cells adjacent to somites 1–5 form the ENS along the entire length of the gut (Espinosa-Medina et al., 2017). In addition, sacral neural crest cells in the somite 24 are the other origin of the ENS, which only migrate into the hindgut, particularly the post-umbilical gut (Durbec et al., 1996). Once reaching the gastrointestinal tract, neural crest cells would differentiate into EGCs and the ENS neurons. SRY-box transcription factor 10 (SOX10) is one of the specific markers for the progenitors of EGCs and could be detected in the foregut as early as embryonic day 9.5 (E9.5) in the mouse (Anderson et al., 2006; Hoff et al., 2008). Notably, studies have shown that SOX10 is involved in gliogenesis in the gut (Kim et al., 2003). In particular, neural crest cells initially express SOX10 and the tyrosine kinase receptor rearranged during transfection (RET) when migrating into the gut (Schuchardt et al., 1994; Kim et al., 2003), but RET would be downregulated for the generation of EGCs (Young et al., 1999). On the other hand, the SOX10 downregulation and the RET maintenance would designate the differentiation of the ENS neurons (Kim et al., 2003; Lasrado et al., 2017). Brain fatty acid-binding protein (B-FABP) then appears at E11.5 in the progenitors of EGCs (Young et al., 2003), followed by proteolipid protein 1 (PLP1) at E12.5 (Lasrado et al., 2017), S100β at E14.5 (Young et al., 2003), and finally glial fibrillary acidic protein (GFAP) at E16.5 (Rothman et al., 1986). Currently, it is incompletely understood how the specific differentiation of EGCs *vs*. the ENS neurons is precisely controlled. For additional information on the ENS development, several recent reviews are available for reference (Lake and Heuckeroth, 2013; Rao and Gershon, 2018; Boesmans et al., 2021).

EGCs are distinct from other types of glial cells and were initially regarded as astrocyte-like glia in the gut (Gabella, 1972; Jessen and Mirsky, 1983). However, the transcription profile of EGCs shares more similarities with the myelinating glial cells such as Schwann cells and oligodendrocytes than with astrocytes (Rao et al., 2015). Moreover, EGCs have been known for their complex heterogeneity. For example, previous works characterized EGCs in the mouse myenteric plexus into two major subtypes based on cellular morphology, i.e., one with an astrocyte-like appearance and the other with fibrous structures (Hanani and Reichenbach, 1994). Notably, cellular morphology and tissue localization exhibit a strong correlation in the classification of EGCs (Gulbransen and Sharkey, 2012; Seguella and Gulbransen, 2021). Recent studies have further classified the mouse EGCs into four subtypes (Figure 1; Hanani and Reichenbach, 1994; Vanderwinden et al., 2003; Savidge et al., 2007; Badizadegan et al., 2014; Boesmans et al., 2015). Subtype I EGCs are intra-ganglionic star-shaped cells with many irregular branches, resembling astrocytes in the central nervous system. Subtype II EGCs are localized within or at the boundary of the submucosal and myenteric plexus and have long processes projecting along with neuronal axons. Subtype III EGCs are enriched within the lamina propria and also outside the submucosal and myenteric plexus, exhibiting a few processes (e.g., 4–5). Subtype IV EGCs are present within the circular and longitudinal layers of smooth muscles and have a relatively simple bipolar morphology.

Unsurprisingly, the morphological heterogeneity of EGCs could be extended to molecular levels. Studies reported that SOX10, S100β, and GFAP were differentially expressed in different subtypes of EGCs (Boesmans et al., 2015). Also, PLP1 rather than GFAP may represent a universal cellular marker for EGCs in the mouse. In particular, subtype IV EGCs in the mouse colon appear PLP1-positive but GFAP-negative (Rao et al., 2015). Moreover, recent works aided by the single-cell RNA sequencing techniques further elucidated the transcriptional complexity of EGCs (Zeisel et al., 2018; Drokhlyansky et al., 2020). For instance, seven distinct subtypes of EGCs were suggested in the myenteric plexus of the mouse small intestine (Zeisel et al., 2018). On the other hand, EGCs in the adult mouse colon were clustered into three subtypes. In addition, it was reported that EGCs could be categorized into three common and three patient-specific subtypes in the human colon under cancer conditions (Drokhlyansky et al., 2020). However, given the complexity of EGCs at morphological and molecular levels, a consensus on their classification remains to be settled in the research field. In light of this evolving issue, our discussion of the immune functions of EGCs would not try to distinguish specific subtypes.

## Immune Functions of EGCs

**Figure 2:**
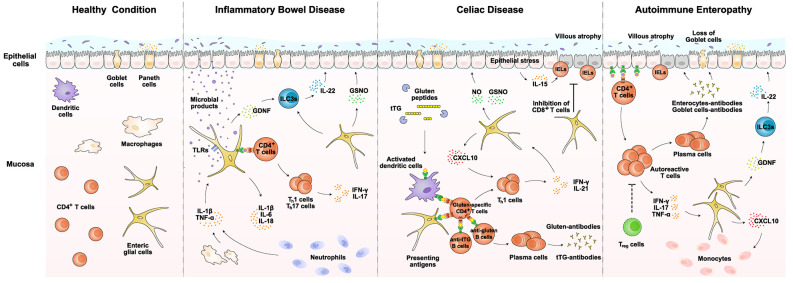
EGCs in immunological disorders of the gut. EGCs exert potential roles in several important immunological disorders of the gut, including inflammatory bowel disease, celiac disease, and autoimmune enteropathy. EGCs could directly sense invading pathogens *via* specific TLRs and release pro-inflammatory cytokines such as IL-1β and IL-6. Also, EGCs are able to produce CXCL10 and facilitate the recruitment of lymphocytes or monocytes. In addition, EGCs may present disease-related antigens *via* MHC-II to CD4+ T cells. Moreover, EGCs would produce GDNF and GSNO that indirectly or directly modulate the epithelial integrity. Details of the immune functions of EGCs are described in the main text. Notably, different subtypes of EGCs are not particularly distinguished for immune functions in this diagram. GDNF, glial cell line-derived neurotrophic factor; GSNO, S-nitrosoglutathione; IELs, intraepithelial lymphocytes; ILC3s, type 3 innate lymphoid cells; NO, nitro oxide; TLRs, Toll-like receptors; tTG, tissue transglutaminase.

EGCs could provide metabolic support to the ENS neurons (Abdo et al., 2010). Also, EGCs may act in the structural preservation of the ENS neurons and their axons during gut motility (Gabella, 1972). In addition, it has been implicated that EGCs might transdifferentiate into the ENS neurons under certain pathological conditions (Joseph et al., 2011; Laranjeira et al., 2011; Belkind-Gerson et al., 2015, 2017). Notably, the immunological aspect of EGCs has recently garnered research attention and emerged as a new frontier topic. We will summarize the known immune functions of EGCs and then discuss their potential roles in several immunological diseases of the gut (Figure 2).

Though EGCs are non-immune cells, they are capable of producing a variety of cytokines and chemokines in response to pathological stimuli. For example, EGCs express Toll-like receptors (TLRs) such as TLR2, TLR3, and TLR4 (Barajon et al., 2009; Turco et al., 2014). Microbial products such as lipopolysaccharide (LPS) could directly activate EGCs to secrete IFN-γ, IL-1β, IL-6, and CCL2 (Murakami et al., 2009; Rosenbaum et al., 2016). Also, EGCs release the macrophage colony-stimulating factor (M-CSF) that regulates the activation of the muscularis macrophages (Grubisic et al., 2020). Further, EGCs would respond to the pro-inflammatory cytokines derived from immune cells such as macrophages and CD4+ T cells. For instance, IL-1β triggers the upregulation of IL-6 and CCL2 in EGCs (Ruhl et al., 2001; Stoffels et al., 2014). Similarly, IFN-γ and LPS co-stimulation of EGCs causes the release of IL-1β and IL-18 (Cirillo et al., 2011; Yang et al., 2020).

EGCs are the predominant cellular source of glial cell line-derived neurotrophic factor (GDNF) in the gut (Meir et al., 2021). GDNF could strengthen the barrier integrity by preventing the apoptosis of intestinal epithelial cells (Savidge et al., 2007; Zhang et al., 2010). Indeed, genetic ablation of GFAP-positive EGCs in mice resulted in fulminant jejunoileitis (Bush et al., 1998), though such observation has been debated by another approach to removing PLP-1-positive EGCs (Rao et al., 2017). In addition, recent works have suggested that such EGCs-derived GDNF would engage type 3 innate lymphoid cells (ILC3s) and stimulate their release of IL-22. This EGCs-GDNF-ILC3s-IL22 axis participates in controlling mucosal homeostasis (Ibiza et al., 2016). At the same time, EGCs could generate S-nitrosoglutathione (GSNO), which acts on epithelial cells to facilitate the formation of tight junctions (Savidge et al., 2007; Cheadle et al., 2013; Li et al., 2016). Therefore, it has become an emerging theme that EGCs play a critical role in modulating the barrier function of the gut *via* such signaling factors (Savidge et al., 2007; Bernardini et al., 2012; Cheadle et al., 2013; Li et al., 2016, 2018).

Besides the release of immune factors, EGCs could directly engage T cell-mediated adaptive immune responses. In particular, EGCs express both MHC-I and MHC-II molecules, enabling them to present disease-related antigens to CD4+ T cells (Geboes et al., 1992; da Silveira et al., 2011; Chow et al., 2021). Also, recent studies reported that EGCs would display inhibitory effects on the proliferation and activity of CD8+ T cells (Kermarrec et al., 2016). Though the mechanism underlying such crosstalk between EGCs and CD8+ T cells remains to be fully elucidated, it has been suggested that EGCs may express the checkpoint blockage protein such as PD-L1, similar to that observed in astrocytes of the central nervous system (Gao et al., 2022). In addition, it appears a possibility that EGCs might influence the B cell-mediated immunity, as EGCs are present in Peyer’s patches (Biskou et al., 2022). Further, EGCs express the immunosuppressive checkpoint protein CD200, which could likely influence T cells, B cells, dendritic cells, and other immune cells in the gut (Chang et al., 2011).

## EGCs in Immunological Disorders

Given the accumulating evidence that EGCs exert immune functions in the gut, their involvement in immunological disorders has begun to emerge (Esposito et al., 2007; von Boyen et al., 2011). In particular, EGCs may participate in disease conditions *via* several mechanisms. First, EGCs could detect invading pathogens and directly elicit the innate immune response (Turco et al., 2014; Rosenbaum et al., 2016). Second, EGCs would respond to pro-inflammatory cytokines derived from other immune cells and augment the local inflammation (Ruhl et al., 2001). Third, EGCs might present disease-related antigens to modulate the gut adaptive immunity (Geboes et al., 1992). Finally, EGCs are able to crosstalk indirectly or directly with the epithelial layer and influence the barrier integrity (Li et al., 2016; Meir et al., 2021). We will focus on the available evidence supporting the roles of EGCs in several gut diseases, i.e., inflammatory bowel disease, celiac disease, and autoimmune enteropathy (Figure 2).

### Inflammatory Bowel Disease

Inflammatory bowel disease (IBD) is one of the most common immunological disorders in the gut. It affects millions of patients globally every year. There are two forms of IBD, i.e., ulcerative colitis and Crohn’s disease (GBD 2017 Inflammatory Bowel Disease Collaborators, 2020). While the underlining causes of IBD vary, it has been recognized that the disease condition is associated with chronic microbial infections, leading to unresolved local inflammation and tissue damage (Halfvarson et al., 2017; Britton et al., 2019). Studies have illustrated that genetic mutations in critical immune signaling components such as nucleotide-binding oligomerization domain containing 2 (NOD2) or IL-18 would predispose the IBD risk (Ogura et al., 2001; Gao et al., 2015). The pathogenic invasion through the epithelial barrier triggers the innate immune responses within the mucosa, resulting in the production of pro-inflammatory cytokines such as IL-1β, IL-6, and TNF-α (Reinecker et al., 1993; Plevy et al., 1997). At the same time, the engagement of CD4+ T cells, particularly Th1 and Th17 cells, would produce IFN-γ and IL-17 (Niessner and Volk, 1995; Fujino et al., 2003). In the normal condition, such a collection of immune actions would eliminate the invading pathogens, enable tissue repair, and restore epithelial integrity. However, in the IBD condition, genetic mutations or other causes would interfere with the effectiveness of immune responses, leading to prolonged inflammation, unresolved infection, and chronic tissue damage (Chang, 2020).

Recent studies have already investigated the role of EGCs in the context of IBD (Bongioanni et al., 1996; Cornet et al., 2001; Cirillo et al., 2009; von Boyen et al., 2011; Bernardini et al., 2012; Li et al., 2018). EGCs could directly detect microbial products such as LPS *via* TLRs that they expressed, which helps elicit the innate immune response together with macrophages (Turco et al., 2014; Rosenbaum et al., 2016). In the meanwhile, EGCs would respond to the macrophage-derived IL-1β and TNF-α and further enhance their production of IL-1β and IL-6 (Ruhl et al., 2001). In addition, EGCs might present microbial-derived antigens to Th1 and Th17 cells, sustaining their production of pro-inflammatory cytokines IFN-γ and IL-17 (Geboes et al., 1992; Chow et al., 2021). Such immune functions of EGCs represent a critical part of local inflammation under the IBD condition. On the other hand, EGCs are capable of promoting barrier integrity and tissue repair. For example, studies reported that EGCs produce GDNF, which acts on ILC3s to stimulate the IL-22 release (Ibiza et al., 2016). IL-22 has an essential role in epithelial regeneration and repair (Lindemans et al., 2015). EGCs also produce GSNO that strengthens the tight junctions between epithelial cells (Li et al., 2016). Therefore, EGCs could exert both pro-inflammatory and tissue-repairing roles in the process of IBD. Indeed, studies reported that the expression levels of GDNF in EGCs correlated with the disease status of patients. Further, the proliferation of EGCs was observed in the condition of ulcerative colitis, probably reflecting their continuous action to counter tissue damage (von Boyen et al., 2011). More detailed examinations of their spatiotemporal response in patients and mouse models of IBD would illustrate the involvement of EGCs in the onset and progression of this common gut disorder.

### Celiac Disease

Celiac disease is another important immunological disorder occurring in the gut. In contrast to the mechanism of IBD, celiac disease is primarily triggered by extrinsic antigens in foods (Ludvigsson et al., 2013; Lebwohl et al., 2018). It affects approximately 1% of the population globally, mainly in Caucasians but relatively rare in Africans and Asians (Singh et al., 2018). Celiac disease is often encountered by the food intake of gluten-containing products (Lebwohl et al., 2018). Gluten is a protein broadly existing in wheat, barley, and other grains. Because gluten highly enriches proline and glutamine residues, the protein is resistant to digestive enzymes, resulting in large amounts of incompletely digested peptides in the small intestine (Shan et al., 2002). These gluten peptides penetrate through the epithelial barrier and get into the lamina propria. The tissue transglutaminase (tTG) deamidates those gluten peptides, which would then be presented as antigens by dendritic cells (Molberg et al., 1998). Notably, people carrying the HLA-DQ2/DQ8 alleles of MHC-II would have a more effective antigen-presentation of gluten peptides and, as a result, become susceptible to celiac disease (Molberg et al., 1998; Sollid et al., 2012). Upon activation by the gluten peptides, CD4+ T cells could release pro-inflammatory cytokines such as IFN-γ and IL-21, initiating the local inflammation in the gut (Bodd et al., 2010). Moreover, the gluten-specific CD4+ T cells further induce the generation of anti-gluten or anti-tTG B cells (Mesin et al., 2012; du Pré and Sollid, 2015). The antibodies targeting gluten or tTG elicit the humoral immune response, particularly in the small intestine (Zanoni et al., 2006; Cervio et al., 2007). In fact, the anti-tTG autoimmune antibodies are the diagnostic hallmark of celiac disease (Lebwohl et al., 2018). In addition, the ongoing inflammation in the lamina propria induces the phenomenon of epithelial stress. In this pathological event, epithelial cells would produce IL-15, which enhances the recruitment of intraepithelial lymphocytes (IELs; Malamut et al., 2010). IELs could compromise the survival and functions of epithelial cells, resulting in the occurrence of villous atrophy (Meresse et al., 2004). Such aberrant immune responses collectively contribute to the chronic pathology observed in celiac disease. This immunological disorder may even proceed to extra-intestinal manifestations such as type I diabetes (Elfstrom et al., 2014).

Research has begun to explore the functional involvement of EGCs in celiac disease (Esposito et al., 2007). Because EGCs are known to express MHC-II molecules in several other disease conditions (Geboes et al., 1992; da Silveira et al., 2011), it appears plausible that they might present the gluten peptides and help trigger CD4+ T cells in the lamina propria, though this possibility remains to be investigated in detail. In addition, EGCs would respond to pro-inflammatory cytokines such as IFN-γ secreted by Th1 cells and upregulate the expression of CXCL10. CXCL10 may then recruit more T cells into the disease-inflicted regions (Progatzky et al., 2021). At the same time, EGCs could exhibit the disease-alleviating roles in celiac disease. For instance, studies reported that EGCs from patients with Crohn’s disease inhibited CD8+ T cells (Kermarrec et al., 2016). Such immunosuppressive function of EGCs on CD8+ T cells might act to ameliorate the damage to the epithelial barrier in celiac disease. In addition, EGCs produce GDNF that acts *via* the aforementioned ILC3s-IL22 mechanism to facilitate tissue repair (Ibiza et al., 2016). Further, EGCs could upregulate the expression of inducible nitric oxide synthase (iNOS) in response to S100β, leading to the production of nitric oxide (NO; Esposito et al., 2007). NO and GSNO together function to restore the barrier integrity of the epithelial layer (Li et al., 2016). Future research would document the precise roles of EGCs in celiac disease, which could open up a new dimension in understanding this immunological disorder of the gut.

### Autoimmune Enteropathy

Similar to celiac disease, autoimmune enteropathy is a gastrointestinal autoimmune disorder. It primarily affects newborns but also occurs in adulthood. The pathological features of this autoimmune disease include severe villous atrophy and the accumulation of intraepithelial CD8+ T cells, resembling those observed in celiac disease (Masia et al., 2014). However, in contrast to celiac disease, autoimmune enteropathy exhibits profound infiltration of monocytes in the lamina propria (Masia et al., 2014). In addition, autoimmune enteropathy has widespread manifestations in extra-intestinal organs, e.g., liver, thyroid, kidney, lung, and skin. The common cause of autoimmune enteropathy is associated with genetic mutations of signaling components involved in the immune tolerance, including forkhead box protein P3 (FoxP3) and the autoimmune regulator (AIRE; Montalto et al., 2009; Chen et al., 2020). Such genetic defects result in the aberrant reaction to self-antigens exposed by epithelial cells or goblet cells. For instance, FoxP3 is an essential transcription factor for the differentiation and functions of regulatory T cells (Treg), which produce the key anti-inflammatory cytokine IL-10 (Hori et al., 2003). Conversely, the loss of FoxP3 abrogates the Treg-mediated immune homeostasis, leading to the appearance of autoimmune CD4+ T cells and B cells in the intestinal tissues and related lymphoid organs (Barzaghi et al., 2012). The autoimmune CD4+ T cells would trigger the recruitment of monocytes (Masia et al., 2014). Moreover, the autoimmune antibodies specific to enterocytes or goblet cells attack the epithelial layer, causing the destruction of barrier integrity and defects in mucus production (Moore et al., 1995; Carroccio et al., 2003).

Though research on the involvement of EGCs in autoimmune enteropathy is still incoming, it is highly possible that EGCs contribute to this disease condition. For example, EGCs release CCL2 or CXCL10 under the LPS or IFN-γ stimulation, which probably facilitates the monocyte recruitment into the lamina propria (Rosenbaum et al., 2016; Progatzky et al., 2021). Further, similar to the scenarios described above, EGCs would maintain the barrier integrity *via* the indirect GDNF-ILC3s-IL22 axis or the direct production of GSNO (Savidge et al., 2007; Ibiza et al., 2016). Future investigations are warranted to unravel the relevance of EGCs in this specific autoimmune disorder of the gut.

### Other Immunological Conditions

Besides those immunological disorders in the gut, EGCs might also participate in the immune responses under divergent pathological conditions. Studies suggested that GDNF and GSNO derived from EGCs could be critical for the maintenance of barrier integrity by upregulating tight-junction proteins during the rotavirus infection (Hagbom et al., 2020). Similarly, the expression levels of GDNF would be upregulated in the colon tissues infected by *Clostridium difficile* (von Boyen et al., 2011). Therefore, it appears possible that GDNF released by EGCs may exert a general protective role in various disease conditions of the gastrointestinal tract. In addition, studies reported that the EGCs expression of MHC-II and the co-stimulatory molecules CD80 and CD86 significantly increased in Chagas disease caused by *Trypanosoma cruzi*, likely contributing to the activation of CD4+ T cells (da Silveira et al., 2011). Therefore, it has become conceivable that EGCs would act in various immunological conditions in the gut, exerting both pro-inflammatory and tissue-repairing functions.

## Future Perspectives

Studies in the past decades have begun to elucidate the critical immune functions of EGCs and their potential involvement in immunological disorders of the gastrointestinal tract. While the research field has witnessed significant advances in the biology of EGCs, we would like to highlight several essential directions that remain to be investigated:

**(1) Comprehensive Documentation of EGCs Immune Capacity**. Studies have already revealed the immunological aspects of EGCs. However, the comprehensive assessment of cytokines, chemokines, and other immune factors that EGCs could produce is still lacking. In addition, the EGCs expression profile of specific receptors for pattern-associated molecular patterns or damage-associated molecular patterns awaits to be defined. Further, whether EGCs could directly present antigens *via* MHC-II molecules to activate CD4+ T cells needs to be unequivocally proven. Research into these questions would chart out the immune capacity of EGCs.

**(2) Pathological Alterations of EGCs**. Notably, the majority of available studies characterized EGCs in normal, healthy conditions. On the other hand, research has begun to reveal that EGCs could undergo significant alterations in the context of different diseases (Linan-Rico et al., 2016; Rosenbaum et al., 2016; Delvalle et al., 2018). For example, EGCs showed a higher expression of interferon-stimulated genes in the conditions of helminth infection or ulcerative colitis (Progatzky et al., 2021). In addition, it was proposed that EGCs could be defined as reactive based on their increased expression of pro-inflammatory pathways (Linan-Rico et al., 2016). Would any new subtype of EGCs emerge in response to specific pathological cues? Would EGCs experience cell death and cell regeneration in the disease-inflicted tissue? These unanswered questions could represent a new dimension in understanding the biology of EGCs.

**(3) EGCs Crosstalk With Other Systems**. While our current discussion has been centered on the interplay between EGCs and immune cells, EGCs could also interact with other systems within the gut. For example, recent studies reported that the ENS neurons would instruct specific immune responses (Veiga-Fernandes and Mucida, 2016; Wang et al., 2022). As a result, EGCs might indirectly exert immunomodulatory functions by influencing the activity of such ENS neurons (De Giorgio et al., 2012; Gulbransen and Sharkey, 2012). In addition, GSNO and NO have been known to cause vasodilation (Laskin et al., 1994). By producing such chemicals, EGCs may facilitate immune cell infiltration. Detailed studies of the functional interactions between EGCs with the ENS, the vascular system, and the lymphatic system would help uncover the complexity underlying tissue homeostasis in the gut.

**(4) EGCs as a Therapeutic Target**. Given their critical roles in sustaining the functions of the ENS neurons, EGCs have already been regarded as a novel therapeutic target. In light of the emerging immunological aspects of EGCs, it becomes even more appealing that EGCs could be exploited for their disease-alleviating actions. For instance, enhancing the GDNF production from EGCs could benefit tissue repair in different diseases of the gastrointestinal tract (Steinkamp et al., 2012; Hagbom et al., 2020). Also, EGCs might be manipulated to present tumor-related antigens to facilitate anti-cancer immunity. Exploring such therapeutic potential of EGCs would promote the basic and translational research of this unique glial type in the gut.

In sum, we have reviewed the updated knowledge of EGCs in several immunological disorders of the gastrointestinal tract. While EGCs are emerging as a new frontier in the research field, critical questions have called for future research efforts. Our in-depth understanding of the immune functions of EGCs may provide valuable insight for treating important human diseases.

## Author Contributions

CL and JY wrote the manuscript. All authors contributed to the article and approved the submitted version.

## Conflict of Interest

The authors declare that the research was conducted in the absence of any commercial or financial relationships that could be construed as a potential conflict of interest.

## Publisher’s Note

All claims expressed in this article are solely those of the authors and do not necessarily represent those of their affiliated organizations, or those of the publisher, the editors and the reviewers. Any product that may be evaluated in this article, or claim that may be made by its manufacturer, is not guaranteed or endorsed by the publisher.
